# Comparison of the Virome of Quarantined Sugarcane Varieties and the Virome of Grasses Growing near the Quarantine Station

**DOI:** 10.3390/v13050922

**Published:** 2021-05-16

**Authors:** Jean H. Daugrois, Denis Filloux, Charlotte Julian, Lisa Claude, Romain Ferdinand, Emmanuel Fernandez, Hugo Fontes, Philippe C. Rott, Philippe Roumagnac

**Affiliations:** 1 CIRAD, UMR PHIM, 34090 Montpellier, France; jean-heinrich.daugrois@cirad.fr (J.H.D.); denis.filloux@cirad.fr (D.F.); charlotte.julian@cirad.fr (C.J.); claude.lisa83@hotmail.fr (L.C.); romain.ferdinand@cirad.fr (R.F.); emmanuel.fernandez@cirad.fr (E.F.); philippe.rott@cirad.fr (P.C.R.); 2 PHIM Plant Health Institute, Univ Montpellier, CIRAD, INRAE, Institut Agro, IRD, 34090 Montpellier, France; 3 Tour du Valat, Research Institute for the Conservation of Mediterranean Wetlands, 13200 Arles, France; fontes@tourduvalat.org; 4 Institut Méditerranéen de Biodiversité et Ecologie, UMR CNRS-IRD, Avignon Université, Aix-Marseille Université, IUT d’Avignon, 337 chemin des Meinajariés, Site Agroparc BP 61207, 84911 Avignon, France

**Keywords:** viral metagenomics, plant quarantine, sugarcane

## Abstract

Visacane is a sugarcane quarantine station located in the South of France, far away from sugarcane growing areas. Visacane imports up to 100 sugarcane varieties per year, using safe control and confinement measures of plants and their wastes to prevent any risk of pathogen spread outside of the facilities. Viruses hosted by the imported material are either known or unknown to cause disease in cultivated sugarcane. Poaceae viruses occurring in plants surrounding the quarantine glasshouse are currently unknown. These viruses could be considered as a source of new sugarcane infections and potentially cause new sugarcane diseases in cases of confinement barrier failure. The aim of this study was to compare the plant virome inside and outside of the quarantine station to identify potential confinement failures and risks of cross infections. Leaves from quarantined sugarcane varieties and from wild Poaceae growing near the quarantine were collected and processed by a metagenomics approach based on virion-associated nucleic acids extraction and library preparation for Illumina sequencing. While viruses belonging to the same virus genus or family were identified in the sugarcane quarantine and its surroundings, no virus species was detected in both environments. Based on the data obtained in this study, no virus movement between quarantined sugarcane and nearby grassland has occurred so far, and the confinement procedures of Visacane appear to be properly implemented.

## 1. Introduction

Sugarcane (*Saccharum* interspecific hybrids) belongs to the Poaceae family and is cultivated in tropical and subtropical areas for the production of sugar and ethanol. To be competitive on the global market and for diversification of products, sugarcane farmers need to use the best-performing varieties and breeders need genetic resources to create them. Consequently, moving plants across international borders for exchange between breeders or directly to producers is an essential step of this process. Plant movement involves biosafety to protect local production by avoiding pathogen introduction and spread. Safe and accurate quarantine procedures need to be implemented, including accurate disease testing [1].

To control disease introduction through sugarcane germplasm movement in the outermost French European regions and for partners in Africa, a sugarcane quarantine station was established in 1971 by the Institut de Recherche en Agronomie Tropicale in Nogent sur Marne, France [2]. This quarantine station moved to Montpellier in 1978 and became Cirad’s sugarcane quarantine in 1984. In 2010, the sugarcane quarantine service was named Visacane (http://visacane.cirad.fr/, accessed on 31 March 2021). Nowadays, Visacane works with research organizations and sugarcane companies from tropical and subtropical locations around the world [3]. It is a go-through sugarcane quarantine located in the South of France, far away from sugarcane-growing areas. The major objective of Visacane is to supply sugarcane germplasm free from potentially damaging pathogens [3]. Viruses are the most detected pathogens in sugarcane quarantine [3,4]. Imported planting material can host viruses [4,5,6] that are either known for damaging sugarcane crops (the overall majority of sugarcane mastreviruses, poaceviruses, poleroviruses, and potyviruses) or whose effect on sugarcane growth is unknown (ampeloviruses, badnaviruses, and at least one umbravirus and one mastrevirus) [4,6,7,8,9]. On the other hand, plant viruses occurring in Poaceae growing around a quarantine glasshouse are usually unknown. If virus-infected, these plants are potential sources of new infections in quarantine in case of passing of viruses through the confinement barriers.

Because Visacane is located outside a sugarcane growing area, the risk of sugarcane infection by surrounding local viruses should be nonexistent or minimal. Nevertheless, the purpose of quarantining is also to avoid the release or escape from quarantine of viruses that could potentially threaten the local vegetation. Infrastructures and processes are rarely 100% safe and a flow may exist between a quarantine glasshouse and its surrounding environment. Importation of infected plants in a new area is a risk for disease emergence and newly introduced viruses may invade communities of native plants [10]. Additionally, virus species infecting several plant species of a same plant family is a common feature, especially in the Poaceae family. For example, maize, sorghum, sugarcane, and several other grasses can host sugarcane mosaic virus [11]. Maize yellow mosaic virus, a polerovirus initially described in maize, was also recently found in sugarcane in Nigeria and China [12,13]. Sorghum mosaic virus, a potyvirus from sorghum, is the causal agent of sugarcane mosaic in the USA [14] and in China [15]. Grassland or wild plants can also serve as reservoirs for known pathogenic viruses such as wheat streak mosaic virus in the Czech Republic [16] or for new emerging plant viruses as illustrated by the epidemic of maize streak virus in Africa [17]. Wild Poaceae from different grasslands host viruses from the genera *Mastrevirus* (*Geminiviridae* family), *Tritimovirus* (*Potyviridae* family) and *Luteovirus* (*Luteoviridae* family) [18,19,20]. Among these, viruses from the genera *Polerovirus* (*Luteoviridae*), *Potyvirus* (*Potyviridae*) and *Mastrevirus* (*Geminiviridae*) cause diseases in sugarcane [8].

Consequently, to investigate the potential flow of plant viruses between a quarantine and its environment, deciphering the plant virome of both entities is necessary. At the present time, the best methodology for virome identification in a specific environment is viral metagenomics, and different approaches are available [21,22]. Virion-associated nucleic acid (VANA) metagenomics has already been used to identify known and new viruses in sugarcane [5,7,23] and in wild Poaceae [24,25,26]. The objective of this study was to use this methodology for the characterization and comparison of the virome of sugarcane varieties located at the quarantine glasshouse of Visacane and the virome of wild Poaceae present in the surrounding environment of this sugarcane quarantine station.

## 2. Material and Methods

### 2.1. Quarantined Sugarcane Sampling

The youngest fully developed leaf (also called the top visible dewlap leaf or TVD leaf) was sampled from 20 sugarcane plants representing 19 diseased varieties currently maintained in the quarantine glasshouse of Visacane at Cirad in Montpellier (Table 1). Five grams of tissue from the bottom part of each leaf blade were cut in small pieces (approximately 2 × 2 mm) with sterilized instruments and stored in plastic bags at −80 °C until further use.

### 2.2. Wild Poaceae Sampling

**Figure 1:**
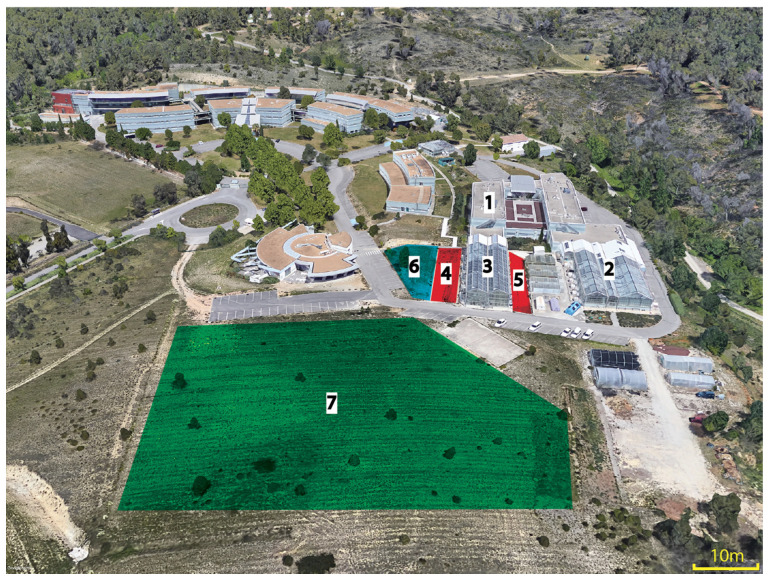
Aerial view of Cirad’s campus showing the sugarcane quarantine (Visacane) and the sampling locations used for analysis of the virome of Poaceae in the environment of the sugarcane quarantine facilities. 1, Research laboratories; 2, plant pathology glasshouse; 3, sugarcane quarantine glasshouse; 4 and 5, flat stone land (framed in red) adjacent to the quarantine glasshouse; 6, sloped land (framed in blue) near the quarantine glasshouse, and 7, grassland (framed in green) near the quarantine glasshouse. This image was retrieved from Google Earth.

Wild Poaceae were collected from three areas surrounding the quarantine glasshouse, including a flat stone land adjacent to the quarantine glasshouse, a sloped land slightly further from the quarantine glasshouse, and a grassland at least 25 m distant from the glasshouse (Figure 1). One hundred and thirty plants representing 25 wild Poaceae species were collected in these three areas (Table 1). A local botanical expert (co-author Hugo Fontes) confirmed the identity of these species, named in accordance with the Euro+Med PlantBase (https://www.emplantbase.org/home.html, accessed on 31 March 2021). Five grams of leaf and stem tissue from each wild Poaceae were cut in small pieces and stored as described above for sugarcane.

### 2.3. Virion-Associated Nucleic Acid-Based Viral Metagenomics

Each of the 150 collected samples was processed using the virion-associated nucleic acid (VANA)-based viral metagenomics approach [27]. Briefly, 1 g of frozen leaf tissue was ground in Hanks’ buffered salt solution (HBSS) (1:10) with four ceramic beads (MP Biomedicals, USA) using a tissue homogenizer (MP biomedicals, USA). The homogenized plant extracts were centrifuged and supernatants were filtered through a 0.45 µm filter and centrifuged at 148,000× *g* for 2.5 h at 4 °C to concentrate viral particles. Nonencapsidated nucleic acids were eliminated by DNase and RNase incubation at 37 °C for 1.5 h. Total RNA and DNA was then extracted using the NucleoSpin kit (Macherey Nagel). Reverse transcription was performed with the SuperScript III reverse transcriptase (Invitrogen), cDNAs were purified with the QIAquick PCR Purification Kit (Qiagen) and complementary strands were synthesized using the Klenow DNA polymerase I. Double-stranded DNA was amplified by random PCR amplification. Samples were barcoded during reverse transcription and PCR steps were performed using homemade 26-nt Dodeca Linkers and PCR multiplex identifier primers. PCR products were purified using NucleoSpin gel and PCR clean-up (Macherey Nagel). Wild Poaceae and sugarcane samples were sequenced in two independent Illumina HiSeq runs in order to avoid index-hopping contamination [28]. Bioinformatics analyses were performed as described previously [27]. Briefly, demultiplexing was performed with the agrep command-line tool to assign reads to the samples from which they originated [29]. Adaptors were removed and the reads were filtered for quality (q30 quality and read length > 45 nt) using Cutadapt 1.9 [30]. The cleaned reads were assembled de novo into contigs using SPAdes 3.6.2 [31]. Putative virus reads and plant virus reads obtained using either BLASTn or BLASTx [32] from sugarcane and wild Poaceae with e-values < 0.001 were retained. Finally, only putative plant viruses with more than 10 reads per sample were taken into consideration for further analysis. Cleaned reads have been deposited in the sequence read archive of GenBank (accession number PRJNA721112).

### 2.4. Partial Genomic Characterization of a Novel Sugarcane Umbravirus

RT-PCR reactions were carried out to extend the genomic sequence of a novel umbravirus isolated from sugarcane variety BJ790038, for which four short contigs (131–325 nt) were recovered using the VANA-based metagenomics approach. PCR reactions were performed using the Qiagen OneStep RT-PCR Kit. Ten primers were designed (Table S1) based on the four contig sequences. The 25 μL RT-PCR reaction mix consisted of 1 μL of eluted RNA, 14.5 μL of RNase-free water, 1µl of RNase inhibitor (RNase-Out, Invitrogen), 5 μL of RT-PCR buffer (5X), 0.5 μL of dNTPmix (10 mM), 1 μL of each primer (10 μM), and 1 μL of the RT-PCR enzyme mix. The RT-PCR program was as follows with the extension time (Ext) for each primer pair listed in Table S1: 50 °C for 30 min, 95 °C for 15 min, 35 cycles at 94 °C for 1 min, annealing temperature 55 °C for 1 min, and 72 °C for Ext with a final 72 °C extension for 10 min. PCR products were analyzed by electrophoresis using a 1.2% agarose gel in TAE buffer stained with ethidium bromide and visualized under UV light. Amplification products were sequenced using the Sanger method (Genewiz, Leipzig, Germany).

### 2.5. Phylogenetic Analyses

Contigs produced by assembly of Illumina reads were utilized as queries to perform BLASTn and BLASTx searches [32] using the NCBI database (https://blast.ncbi.nlm.nih.gov/Blast.cgi, accessed on 31 March 2021). Forty-eight contigs assigned to plant viruses obtained from both compartments (within and outside the quarantine) were selected for further comparisons. These 48 sequences were subsequently aligned to reference genomes using ClustalW with default settings [33]. Visual inspection of alignment quality and BLAST graphic summaries was done and one contig presenting one chimeric end was trimmed. The resulting alignments were used to infer neighbor-joining phylogenetic trees using the MegaX software [34]. The Jukes-Cantor nucleotide substitution model with 1000 bootstrap replicates for branch support was applied. The 48 contigs obtained from quarantined sugarcane varieties and wild Poaceae samples, assigned to the *Caulimoviridae, Closteroviridae, Geminiviridae, Luteoviridae, Potyviridae,* and *Tombusviridae* families, that were used for the phylogenetic analyses described above are listed in the Supplementary Material.

## 3. Results

### 3.1. Analysis of the Virome of Quarantined Sugarcane and of Wild Poaceae Growing Outside the Sugarcane Quarantine Glasshouse

Total numbers of Illumina reads obtained for each of the 20 samples of quarantined sugarcane varieties varied from 125,826 (FR95433) to 625,654 (KN8924), with an average number of 315,508 reads per sample (Table 1). Retained virus reads represented 24% of total reads whereas the 1483,466 plant virus reads represented 97.6% of the virus reads (Table 2), with an average of 74,173 plant virus reads per quarantined sugarcane sample. The number of reads per putative plant virus family ranged from 1339 (*Tombusviridae*) to 833,570 (*Geminiviridae*) (Table 2).

Total numbers of Illumina reads obtained for each of the 130 samples of wild Poaceae collected outside the sugarcane quarantine varied from 40 (*Helictochloa bromoides* from grassland) to 414,878 (*Bromopsis erecta* from flat stone land), with an average number of 175,553 reads per sample. Retained virus reads represented 2.8% of the 22,821,881 reads obtained for the three sampled locations (Table 1), whereas the 69,065 plant virus reads represented 10.9% of the virus reads, with an average of 531 plant virus reads per wild Poaceae sample. The overall majority of viral sequences were assigned to viruses infecting bacteria, fungi, and arthropoda (Table 2).

Contigs from quarantined sugarcane varieties were assigned to seven virus families, i.e., *Alphasatellitidae, Caulimoviridae, Closteroviridae, Geminiviridae, Luteoviridae, Potyviridae,* and *Tombusviridae* (Table 2). With the exception of 11 contigs, the contigs obtained from the quarantined sugarcane varieties were all assigned to sugarcane viruses already known to infect the sugarcane plants maintained in the quarantine glasshouse of Visacane at Cirad in Montpellier. The 11 contigs that were not assigned to a known sugarcane virus were produced from two quarantined sugarcane varieties and all shared highest identity with umbraviruses (see below). Plant virus contigs obtained from wild Poaceae samples were distributed into 11 plant virus families (*Amalgaviridae*, *Aspiviridae,* *Bromoviridae*, *Caulimoviridae*, *Closteroviridae****,*** *Endornaviridae*, *Geminiviridae*, *Luteoviridae*, *Potyviridae, Retroviridae,* and *Tombusviridae*)*,* one unclassified genus *(Sobemovirus)* and one unclassified plant-associated virus, Trifolium-associated circular DNA virus (Table 2). Contigs assigned to *Closteroviridae*, *Caulimoviridae, Geminiviridae, Luteoviridae, Potyviridae* and *Tombusviridae* families were each produced from wild Poaceae samples and from quarantined sugarcane samples. Specifically, contigs assigned to the *Badnavirus* (*Caulimoviridae*), *Mastrevirus* (*Geminiviridae*) and *Umbravirus* (*Tombusviridae*) genera were present in both quarantined sugarcane samples and wild Poaceae samples.

### 3.2. Phylogenetic Relationships of Plant Viral Sequences Assigned to the Closteroviridae, Luteoviridae, and Potyviridae Families

**Figure 2:**
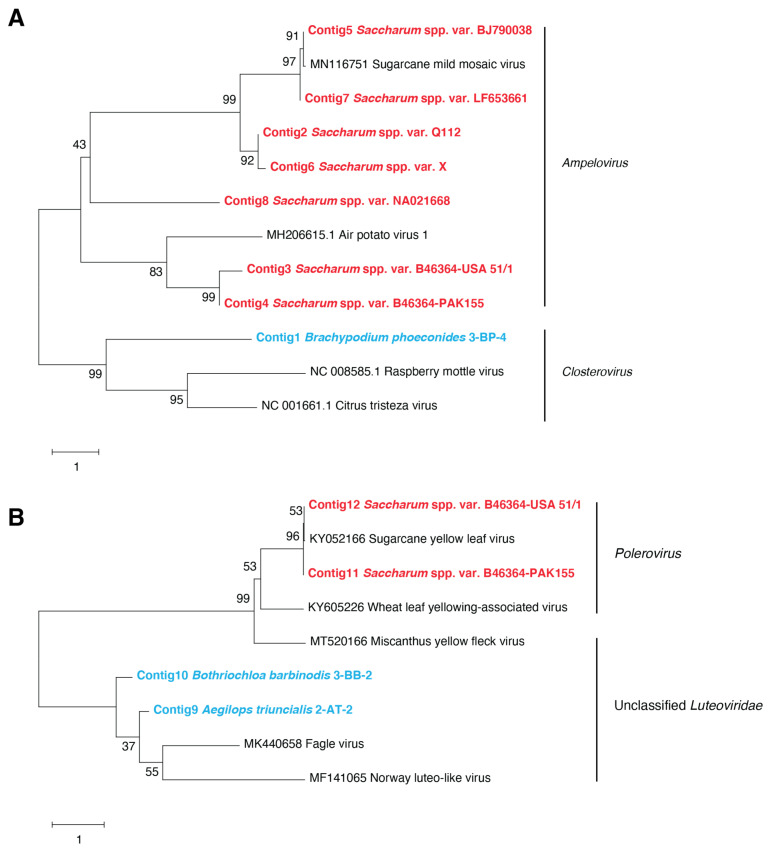
(**A**) Neighbor-joining phylogenetic tree of *Closteroviridae* contigs obtained from one wild Poaceae species (bold characters colored in blue), sugarcane varieties (bold characters colored in red), and representative closteroviruses and ampeloviruses genome sequences (colored in black). (**B**) Neighbor-joining phylogenetic tree of *Luteoviridae* contigs obtained from two wild Poaceae species (bold characters colored in blue), two plants from one sugarcane variety (bold characters colored in red), and representative *Luteoviridae* genome sequences (colored in black). Scale bars represent the number of substitutions per site; branch labels correspond to the consensus support (%). The 12 contigs produced from quarantined sugarcane varieties and wild Poaceae samples, assigned to the *Closteroviridae* and *Luteoviridae* families, are listed in the Supplementary Material.

While the 101 *Closteroviridae* contigs obtained from seven quarantined sugarcane varieties (Q112, B46364-USA51/1, B46364-PAK155, BJ790038, X, LF653661, and NA021668) were assigned to the *Ampelovirus* genus, one *Closteroviridae* contig obtained from a grassland sample of *Brachypodium phoenicoides* was assigned to the *Closterovirus* genus using BLASTx searches (Figure 2A and Table S2). The highest identity score for this 535-nt-long contig obtained from *B. phoenicoides* was obtained with raspberry leaf mottle virus (accession number QOS14265, highest percent identity = 36%, e-value = 3 × 10^−10^), suggesting that it represented a novel closterovirus that needs further characterization. The phylogenetic analyses also showed that the sugarcane *Ampelovirus* contigs grouped together but were also distributed into several subgroups, thus indicating that sugarcane-associated ampeloviruses are diverse and may represent several divergent variants of the same species, or even several species (Figure 2A).

Two contigs from an *Aegilops triuncialis* plant, one contig from a *Bothriochloa barbinodis* plant, and two large contigs from two sugarcane varieties (B46364-USA51/1 and B46364-PAK155) were assigned to the *Luteoviridae* family using BLAST searches (Table S3). The large contig from each sugarcane variety shared 99% identity with sugarcane yellow leaf virus (SCYLV, accession number KY052166) and represented 97.7% of the complete genome sequence of this SCYLV isolate. The two overlapping contigs from *A. triuncialis* and *B. barbinodis* shared 71% identity among each other, suggesting that they could be variants of the same virus. Identities of the three wild Poaceae contigs with SCYLV (accession number KY052166) ranged from 39 to 43% and from 70.8 to 74.9% with unclassified luteoviruses isolated from insects (Fagle virus, accession number MK440658 and Norway luteo-like virus 1, accession number MF141065). The phylogenetic analysis allowed us to confirm that the *Luteoviridae* contigs obtained from the two wild grasses were highly divergent from plant luteoviruses (Figure 2B).

**Figure 3:**
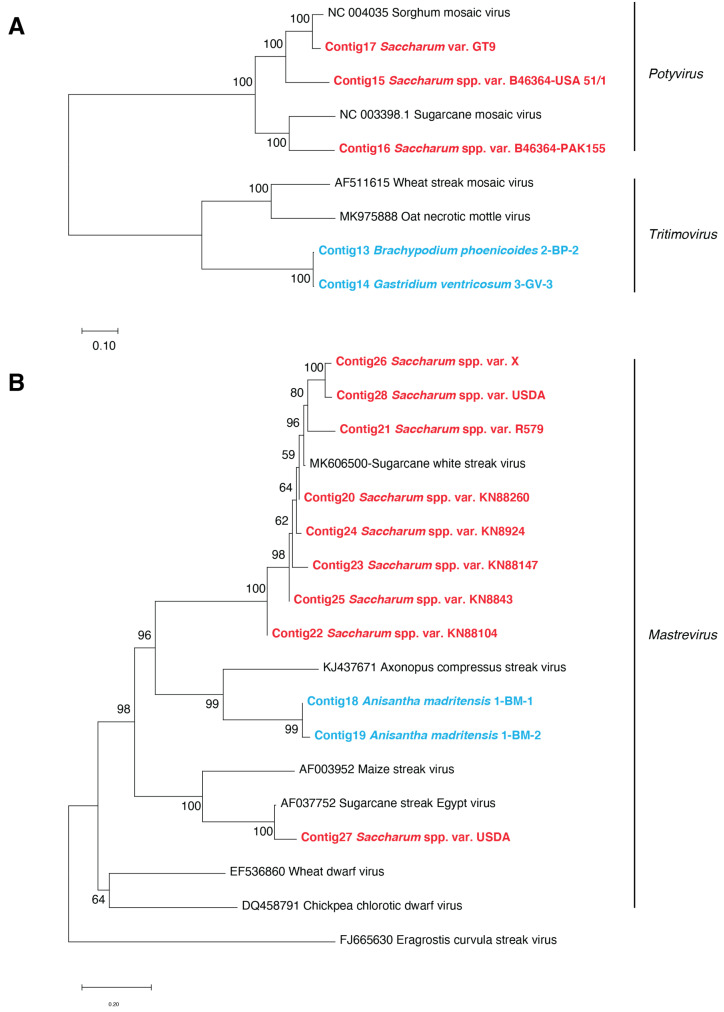
(**A**) Neighbor-joining phylogenetic tree of *Potyviridae* contigs obtained from wild Poaceae (bold characters colored in blue), sugarcane varieties (bold characters colored in red), and representative potyviruses and tritimovirus genome sequences (colored in black). (**B**) Neighbor-joining phylogenetic tree of *Geminiviridae* contigs obtained from wild Poaceae species (bold characters colored in blue), sugarcane varieties (bold characters colored in red), and representative mastrevirus genome sequences (colored in black). Scale bars represent the number of substitutions per site; branch labels correspond to the consensus support (%). The 16 contigs obtained from quarantined sugarcane varieties and wild Poaceae samples, assigned to the *Potyviridae* and *Geminiviridae* families, are listed in the Supplementary Material.

Complete genome sequences of viral isolates belonging to the *Potyviridae* family were obtained from both quarantined sugarcane varieties and wild Poaceae samples. Three complete genome sequences, each produced from a different sugarcane variety, were assigned to the genus *Potyvirus*. On the other hand, two complete genome sequences obtained from two wild Poaceae samples were assigned to the genus *Tritimovirus* (Table S4). These results confirmed the perennial conservation of two potyviruses (sorghum mosaic virus (SrMV) isolate USA51/1 and sugarcane mosaic virus (SCMV) isolate PAK155) that were inoculated in 2002 in Montpellier on two plants of sugarcane variety B46364. The complete genome sequence obtained from sugarcane variety GT9 was also phylogenetically related to sorghum mosaic virus (Figure 3A), and the genome nucleotide sequences of the two virus isolates were 98% identical. The two complete genome sequences recovered from a sloped land sample of *Brachypodium phoenicoides* and a grassland sample of *Gastridium ventricosum* shared 99% nucleotide identity. This suggested that both sequences belonged to the same *Tritimovirus* species. Furthermore, these two Poaceae tritimovirus sequences shared 67.6% nucleotide identity with oat necrotic mottle virus (accession number AY377938), which is below the species demarcation (<76% nucleotide identity) of the *Potyviridae* family [35]. Consequently, these sequences could represent a novel species of the *Tritimovirus* genus.

### 3.3. Phylogenetic Relationships of Plant Viral Sequences Assigned to the Mastrevirus, Badnavirus, and Umbravirus Genera

Mastrevirus contigs were obtained from eight sugarcane varieties and from two *Anisantha madritensis* plant samples collected from the flat stone land (Table S5). One complete genome sequence of sugarcane streak Egypt virus (SSEV) and five complete genome sequences of sugarcane white streak virus (SWSV) were produced from one sugarcane variety (USDA) and from five sugarcane varieties (USDA, R579, X, KN88147, and KN8924), respectively. The five SWSV complete genome sequences shared 91–99% nucleotide identities among each other. Additionally, three partial SWSV genome sequences were obtained from three other sugarcane plant samples (KN88260, KN88104, and KN8843). The complete genome nucleotide sequences that were recovered from the two plants of *A. madritensis* were 99.9% identical. These two sequences had 75% identity with sorghum arundinaceum-associated virus (MK546381), a mastrevirus recently reported from the Réunion Island. They also shared 44.3–46.2% identity with the SSEV and SWSV genome sequences retrieved from the quarantined sugarcane plant samples. In a phylogenetic tree constructed with entire genome sequences of known mastreviruses and those obtained in this study, the two sequences from the wild Poaceae plants were also located at a unique branch (Figure 3B). According to the current species demarcation (<78% nucleotide identity) for the genus *Mastrevirus* [36], the *A. madritensis*-derived mastrevirus is therefore likely to be a novel species.

**Figure 4:**
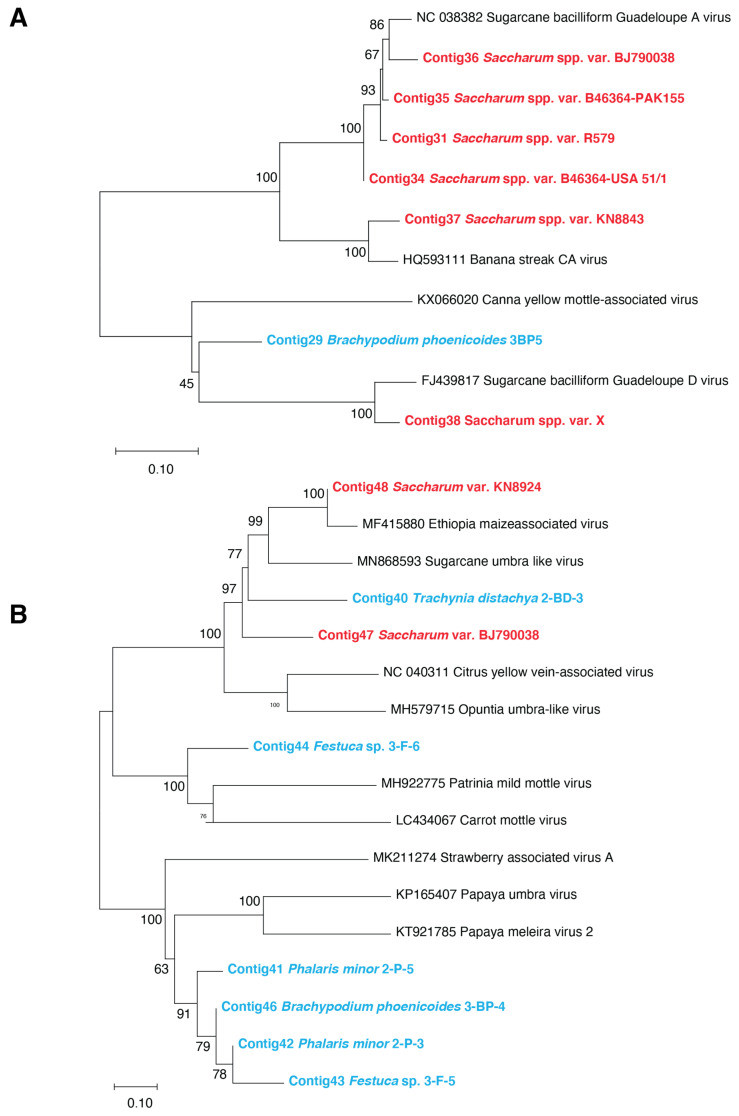
(**A**) Neighbor-joining phylogenetic trees of *Caulimoviridae* contigs obtained from one wild Poaceae species (bold characters colored in blue), sugarcane varieties (bold characters colored in red), and representative badnaviruses genome sequences (colored in black). (**B**) Neighbor-joining phylogenetic trees of *Tombusviridae* contigs obtained from wild Poaceae species (bold characters colored in blue), sugarcane varieties (bold characters colored in red), and representative classified and unclassified umbravirus genome sequences (colored in black). Scale bars represent the number of substitutions per site; branch labels correspond to the consensus support (%). The 15 contigs produced from quarantined sugarcane varieties and wild Poaceae samples, assigned to the *Caulimoviridae* and *Tombusviridae* families are listed in the Supplementary Material.

Eighty-five contigs assigned to the *Badnavirus* genus (*Caulimoviridae*) were obtained from nine sugarcane samples (B46364 USA51/1, B46364 PAK155, BJ790038, KN8843, KN88104, LF653661, Q112, R579, and X), from which two contigs, each covering the full badnavirus genome, were produced for sugarcane variety BJ790038 and variety X (Table S6). For the wild Poaceae, only one sample of *B. phoenicoides* collected from grassland contained four badnavirus contigs (Table S6) that shared 74.5–77% nucleotide identity with canna yellow leaf mottle virus (CaYMV, accession number KX255725) and 30 to 72% nucleotide identity with the badnavirus contigs obtained from quarantined sugarcane varieties. Based on phylogenetic analyses, the two longest contigs of *B. phoenicoides* clustered with CaYMV and formed a group that was apart from any of the badnaviruses currently known to infect sugarcane (Figure 4A and Figure S1) [37]. The contigs produced from sugarcane varieties R579, B46364 USA51/1, B46364 PAK155, and BJ79038 were related to sugarcane baciliform Guadeloupe A virus (SCBGAV, accession number NC_038382) (Figure 4A). The contigs obtained from the variety X clustered with sugarcane bacilliform Guadeloupe D virus (SCBGDV, accession number NC_013455) (Figure 4A and Figure S1) and contigs obtained from varieties KN88104, KN8843, and LF653661 grouped with banana streak CA virus (accession number HQ593111) (Figure 4A and Figure S1). The contigs from sugarcane variety Q112 matched with two different badnavirus genomes, i.e., SCBGAV and sugarcane bacilliform virus isolate Iscam (accession number JN377534) (data not shown).

Contigs assigned by BLAST searches to the *Tombusviridae* family, including members of the *Umbravirus* genus and unclassified umbraviruses, were obtained from five wild Poaceae species and two sugarcane varieties (BJ79038 and KN8924). These wild *Poaceae* samples included *Trachynia distachya*, *Bothriochloa barbinodis, B. phoenicoides*, *Phalaris minor,* and *Festuca* sp. plants from both the sloped land and the grassland areas (Table S7). *Umbravirus* contigs recovered from wild Poaceae plants shared 45–72% nucleotide identities with a sugarcane umbra-like virus genome (accession number MN868593). Four contigs obtained from two *P. minor* plants, one *Festuca* sp. plant, and one *B. phoenicoides* plant had 66–69% nucleotide identity with strawberry-associated virus A (accession number MK211274). The four contigs also clustered in a neighbor-joining phylogenetic tree, thus suggesting that a single novel umbravirus infected these wild Poaceae plants (Figure 4B). Furthermore, while the contig recovered from the other *Festuca* sp. plant shared 71% nucleotide identity with Patrinia mild mottle virus (accession number MH922775), contigs from *T. distachya* and *B. barbinodis* were 100% identical and had 71–73% nucleotide identity with Ethiopia-maize-associated virus (accession number MF415880). Finally, two partial genome sequences of umbraviruses (1931 nt and 2039 nt, Table S7) were recovered from sugarcane varieties (BJ79038 and KN8924), respectively, using VANA-based reads and RT-PCR assays. These two partial genomes were 71% identical. The 2039-nt-long partial genome from the sugarcane variety KN8924 shared 97% nucleotide identity with Ethiopia-maize-associated virus (accession number MF415880), suggesting that this still-unclassified umbra-like virus, initially isolated from maize, was also infecting sugarcane. The 1931-nt-long partial genome from sugarcane variety BJ79038 shared 72% nucleotide identity with Ethiopia-maize-associated virus (accession number MF415880) and 71% with sugarcane umbra-like virus (accession number MN868593).

## 4. Discussion

The plant virome of the wild Poaceae collected near the quarantine glasshouse included 11 virus families, one unclassified genus (*Sobemovirus*), and one unclassified plant-virus (trifolium-associated circular DNA virus). In contrast, only seven families formed the plant virome of the quarantined sugarcane varieties. Most virus sequence reads (85%) obtained from wild Poaceae were attributed to non-plant viruses (bacterial viruses: *Microviridae* viruses, bacteriophages, and caudovirales; bird and mammal viruses: *Circoviridae* viruses and *Genomoviridae* viruses; fungal viruses: viruses belonging to the families *Chrysoviridae*, *Partitiviridae*, *Totiviridae*, and *Genomoviridae*; vertebrate and insect viruses: *Parvoviridae* viruses). Fungal virus reads related to *Chrysoviridae*, *Partitiviridae*, and *Totiviridae* were previously identified in wild plants, including Poaceae [26,38]. The frequency of this type of reads was very low (1%) in quarantined sugarcane samples for which more than 99% of the virus reads were associated to plant viruses, suggesting that plants growing in quarantine are protected from infections by environmental microorganisms. However, six of the seven virus families found in quarantined sugarcane were also represented in wild Poaceae species growing outside the restricted area. These families included *Caulimoviridae*, *Closteroviridae*, *Geminiviridae*, *Luteoviridae*, *Potyviridae*, and *Tombusviridae*. The sequences obtained herein for the *Closteroviridae*, *Luteoviridae*, and *Potyviridae* families belonged to different virus genera when comparing virus isolates from quarantined plants and from plants growing in the outside quarantine environment. Nevertheless, even if the virus genera between quarantined and non-quarantined plants are different, the risk of cross infection cannot be eliminated. For the contigs assigned to the three other families, the genera were common between quarantined sugarcane and wild Poaceae. These common genera were identified as *Badnavirus* (*Caulimoviridae* family), *Mastrevirus* (*Geminiviridae* family), and *Umbravirus* (*Tombusviridae* family).

Major plant species infected by badnaviruses have a tropical or sub-tropical origin (aglaonema, alpinia, banana, bougainvillea, cacao, canna, citrus, codonopsis, dracaena, jujube, kalanchoe, pagoda, pineapple, piper, shefflera, stilbocarpa, sugarcane, sweet potato, taro, wisteria, yacon, yam, and yucca) [39]. Badnaviruses were also reported in Europe in rubus [40], grapevine [41], birch [42], and fig [43], but not in wild Poaceae. Badnaviruses are frequently detected in sugarcane growing in tropical and sub-tropical locations of all continents with relative high genetic variability and numerous species groups [44]. The badnavirus sequences found in *B. phoenicoides* were different but close to the sequence identified in sugarcane variety X that belonged to badnavirus group 1 subgroup D [37], thus indicating potential virus transfer between the sugarcane quarantine in Montpellier and surrounding areas. However, the probability that sugarcane is the source of the badnavirus identified in *B. phoenicoides* near Visacane is very low because the same badnavirus was also found in *B. phoenicoides* samples collected at Villeveyrac (12 reads) and Pellissanne (12 reads), two locations that are, respectively, 30 and 120 km distant from the sugarcane quarantine in Montpellier (Roumagnac, unpublished results). However, further investigations are needed to identify the potential risk for sugarcane to be infected by the putative *B. phoenicoides* badnavirus discovered in this study.

Mastreviruses occur in cultivated and wild Poaceae in several continents [18,45,46,47]. For instance, wild Poaceae host various mastreviruses in Nigeria and are considered as reservoirs for maize streak virus [47]. In Australia, three mastreviruses were identified in wild Poaceae but not in cultivated Poaceae [46]. Up to now, only three mastreviruses have been reported in Europe. Wheat and barley dwarf viruses were initially reported in only a few European countries [48], but were found in additional European countries a few years later [49,50]. A survey undertaken in Germany revealed occurrence of at least three mastreviruses in cultivated Poaceae in this country: barley dwarf virus, oat dwarf virus, and wheat dwarf virus [51]. To our knowledge, the mastrevirus identified in the wild Poaceae *A. madritensis* in our study is the first report of this virus. It could be the cause of new diseases of cultivated Poaceae in Europe. This virus is different from currently known mastreviruses of sugarcane and, although genetically distant from sugarcane streak Egypt virus (SSEV) and sugarcane white streak virus (SWSV), it might be able to cause disease in sugarcane in cases of accidental spread to healthy sugarcane plants.

Umbraviruses and unclassified umbra-like viruses were the final viruses shared by quarantined sugarcane and wild Poaceae. Umbra-like viruses, which are similar to umbraviruses but do not contain all of the umbravirus genomic features [9], have been previously found in sugarcane in Florida [7] and South Africa [9]. Umbra-like virus sequences have, so far, not been reported in other Poaceae with the exception of Ethiopia-maize-associated virus that was initially considered as an unclassified virus [52]. In this study, sequences related to sugarcane and maize umbra-like viruses were clearly identified in sugarcane varieties KN8924 and BJ79038, as well as in wild Poaceae species (*B. barbinodis* and *T. distachya*). Specifically, Ethiopia-maize-associated virus was detected in the quarantine glasshouse in the sugarcane variety KN8924 that originated from Sudan. Consequently, umbra-like virus diversity does not appear related to plant species and this type of virus could spread among different plant hosts, such as between plants in quarantine and in surrounding area compartments. Several other umbravirus isolates found in wild Poaceae in this study differed genetically from isolates close to the sugarcane and maize umbra-like viruses. Isolates of these putative novel umbraviruses were distributed in two phylogenetic subgroups suggesting that two unclassified umbraviruses, not related to any currently known sugarcane umbravirus, were circulating among several wild Poaceae species growing near the sugarcane quarantine greenhouse in the South of France. These two viruses may represent two novel species of the genus *Umbravirus* while the International Committee on Taxonomy of Viruses recommends a species demarcation threshold of <70% nt sequence identity for umbraviruses [53].

## 5. Conclusions

The area surrounding the sugarcane quarantine of Cirad in the South of France hosts a great diversity of viruses in wild Poaceae, which appears greater than the one occurring in quarantined sugarcane varieties. As these two environments share viruses from the same family and even from the same genus, the risk of cross contamination seems higher than expected. However, we showed in this study that no identical or highly similar virus sequences were present in quarantined material and in plants growing in its surrounding environment. This suggested that plant virus movement between quarantined sugarcane and plants of nearby locations has not, so far, occurred. While a large majority of plant viruses are transmitted by insect vectors [54], these results imply that the confinement procedures of Visacane (including insect-proof structures and security portals) appear to be properly implemented.

## Figures and Tables

**Table 1:** List of plant samples processed by virion-associated nucleic acid metagenomics and number of reads obtained for each species/location.

Quarantined Sugarcane		Wild Poaceae from Flat Stone Land		Wild Poaceae from Sloped Land		Wild Poaceae from Grassland		
Variety	Number of Reads	Species	Number of Reads	Species	Number of Reads	Species	Number of Reads	
KN88260	169,914	*Aegilops triuncialis* (3) ^a^	1,228,780	*Aegilops triuncialis* (3)	455,952	*Avena sterilis* (2)	376,016	
R579	337,700	*Aegilops geniculata* (3)	1,051,536	*Aegilops geniculata* (3)	425,570	*Bothriochloa barbinodis* (4)	1,190,788	
KN88104	364,446	*Anisantha madritensis* (6)	1,661,880	*Avena sterilis* (2)	249,804	*Brachypodium phoenicoides* (5)	885,284	
Q112	189,526	*Avena sterilis* (2)	800,428	*Bothriochloa barbinodis* (3)	981,718	*Bromopsis erecta* (3)	1,028,474	
B46364-USA51/1	301,906	*Bothriochloa ischaemum* (6)	931,920	*Trachynia distachya* (2)	374,270	*Cynodon dactylon* (2)	447,196	
KN88147	483,278	*Trachynia distachya* (3)	1,088,638	*Brachypodium phoenicoides* (2)	859,170	*Festuca sp*. (5)	728,278	
BJ79038	244,194	*Bromopsis erecta* (2)	1,102,196	*Bromopsis erecta* (3)	152,906	*Gastridium ventricosum* (6)	1,065,580	
KN8924	625,654	*Bromus lanceolatus* (3)	516,490	*Catapodium rigidum* (2)	681,122	*Helictochloa bromoides* (1)	150,384	
KN8843	391,108	*Catapodium rigidum* (2)	922,976	*Cynodon dactylon* (2)	539,866			
GT9	596,222	*Cynodon dactylon* (2)	52,6150	*Phalaris minor* (6)	1,161,018			
B46364-PAK155	386,752	*Elytrigia intermedia* (6)	1,270,630					
X	269,742	*Hainardia cylindrical* (6)	998,856					
USDA	598,072	*Holcus lanatus* (2)	386,482					
LF653661	189,262	*Hordeum murinum subsp. Leporinum* (6)	806,796					
NA021668	236,036	*Lolium rigidum* (6)	1,353,330					
SP701284	267,242	*Melica ciliata* (6)	520,800					
CR9821	186,898	*Piptatherum miliaceum* (1)	284,228					
Q140	218,214	*Rostraria cristata* (3)	602,560					
FG087484	128,726	*Vulpia ciliata* (6)	570,698					
FR95433	125,826							
Total (samples) reads								
(20)	6,310,178	(74)	12,561,594	(28)	4,388,287	(28)	5,872,000	

^a^ The number in parentheses represents the number of samples collected per plant species.

**Table 2:** Viruses identified in wild Poaceae and quarantined sugarcane varieties by virion-associated nucleic acid metagenomics.

Putative Virus	Virus Host *	Wild Poaceae		Quarantined Sugarcane	
		Number of Samples	Number of Reads	Number of Samples	Number of Reads
*Geminiviridae*	Plant	2	48,512	8	833,570
*Potyviridae*	Plant	2	15,029	3	414,737
*Tombusviridae*	Plant	13	3578	2	1339
*Luteoviridae*	Plant	2	270	2	83,338
*Closteroviridae*	Plant	1	368	7	10,260
*Alphasatellitidae*	Plant			3	140,222
*Amalgaviridae*	Plant	4	657		
*Bromoviridae*	Plant	1	25		
*Aspiviridae*	Plant	1	19		
*Sobemovirus*	Plant	1	20		
*Trifolium-ass.* DNA virus	Plant	3	587		
*Caulimoviridae*	Plant/insect	2	44	9	37,192
*Partitiviridae*	Plant/fungus	89	89,391	5	210
*Endornaviridae*	Plant/fungus/oomycete	2	66		
*Rhabdoviridae*	Plant/vertebrate/invertebrate	4	493		
*Reoviridae*	Plant/fungus/vertebrate/invertebrate	1	413		
*Chrysoviridae*	Fungus	29	4197		
*Narnaviridae*	Fungus	1	15		
*Hypoviridae*	Fungus	6	560		
*Gammaflexiviridae*	Fungus	2	477		
*Metaviridae*	Fungus	2	125		
*Botybirnavirus*	Fungus	21	2554		
Tymovirales (unclassified)	Fungus	2	75		
Unclassified fungal viruses	Fungus	31	35,671		
*Totiviridae*	Fungus/protozoan	40	60,090		
*Genomoviridae*	Fungus/human/mammal/bird	30	6678	1	15
*Microviridae*	Bacteria/spiroplasma	26	35,410		
*Myoviridae*	Bacteria/archaea	5	1419	1	13
*Podoviridae*	Bacteria/archaea	5	6673		
*Siphoviridae*	Bacteria/archaea	5	152,598		
Unclassified bacterial viruses	Bacteria	3	108		
*Iflaviridae*	Insect	1	220		
*Bidnaviridae*	Insect	1	32		
*Parvoviridae*	Insect/vertebrate	10	3271		
*Baculoviridae*	Insect/decapod	1	19	1	18
*Peribunyaviridae*	Insect/rodent	1	16		
Unclassified arthropod viruses	Arthropod	18	20,812		
*Retroviridae*	Vertebrate	2	70		
*Circoviridae*	Bird/mammal	26	8208		
*Iridoviridae*	Amphibia/fish/invertebrate	1	23		
*Phycodnaviridae*	Alga	15	450	1	19
*Mimiviridae*	Amoebae	10	277		
*Nimaviridae*	Crustacean	1	15		
*Arenaviridae*	Rodent	1	19		
unclassified	Vertebrate/invertebrate/crustacean	11	3915		
unclassified	environmental	8	96,793		
unclassified	unknown	6	33,824		
Total reads			634,566		1,520,933

* as reported by ViralZone, SIB Swiss Institute of Bioinformatics.

## Data Availability

Cleaned Illumina reads determined as part of this study have been deposited in the sequence read archive of GenBank (accession number PRJNA721112).

## Supplementary Materials

The following are available online at https://www.mdpi.com/article/10.3390/v13050922/s1, Figure S1: Neighbor-joining phylogenetic trees of Caulimoviridae contigs obtained from sugarcane varieties (bold characters colored in red) and representative badnaviruses genome sequences (colored in black). Scale bars represent the number of substitutions per site; Branch label corresponds to the consensus support (%). Table S1: Primer pairs used for amplification of the partial genome sequence of the umbra-like sequence isolated from sugarcane variety BJ790038, Table S1: Wild Poaceae sample collected from grassland and sugarcane samples from the quarantine glasshouse infected by *Closteroviridae* viruses. Table S3: Wild Poaceae samples collected from the sloped land and the grassland and sugarcane samples from the quarantine glasshouse infected by *Luteoviridae* viruses. Table S4: Wild Poaceae samples collected from the sloped land, the grassland and sugarcane samples from the quarantine glasshouse infected by *Potyviridae* viruses. Table S5: Wild Poaceae samples collected from the flat stone land and sugarcane samples from the quarantine glasshouse infected by mastreviruses (*Geminiviridae* family). Table S6: Selected contig sequences of wild Poaceae samples collected from the grassland and sugarcane samples from the quarantine glasshouse potentially infected by badnaviruses (*Caulimoviridae* family). Table S7: Wild Poaceae samples collected from the sloped land, the grassland and from sugarcane samples located in the quarantine glasshouse, containing *Umbravirus* or umbra-like virus contigs. Contigs in bold were used for phylogenetic analyses. Material S1: Sequences of the 48 contigs obtained from quarantined sugarcane varieties and wild Poaceae samples, assigned to the *Caulimoviridae, Closteroviridae, Geminiviridae, Luteoviridae, Potyviridae,* and *Tombusviridae* families, that were used for the phylogenetic analyses.
