# Supplementary material for: Comparison of the Virome of Quarantined Sugarcane Varieties and the Virome of Grasses Growing near the Quarantine Station
Source: Viruses. 2021 May 16;13(5):922. doi: 10.3390/v13050922 (PMC8157134; doi:10.3390/v13050922)

Supplementary Figure S1. Neighbor-joining phylogenetic trees of Caulimoviridae contigs obtained from sugarcane varieties (bold characters colored in red) and representative badnaviruses genome sequences (colored in black). Scale bars represent the number of substitutions per site; Branch label corresponds to the consensus support (%).

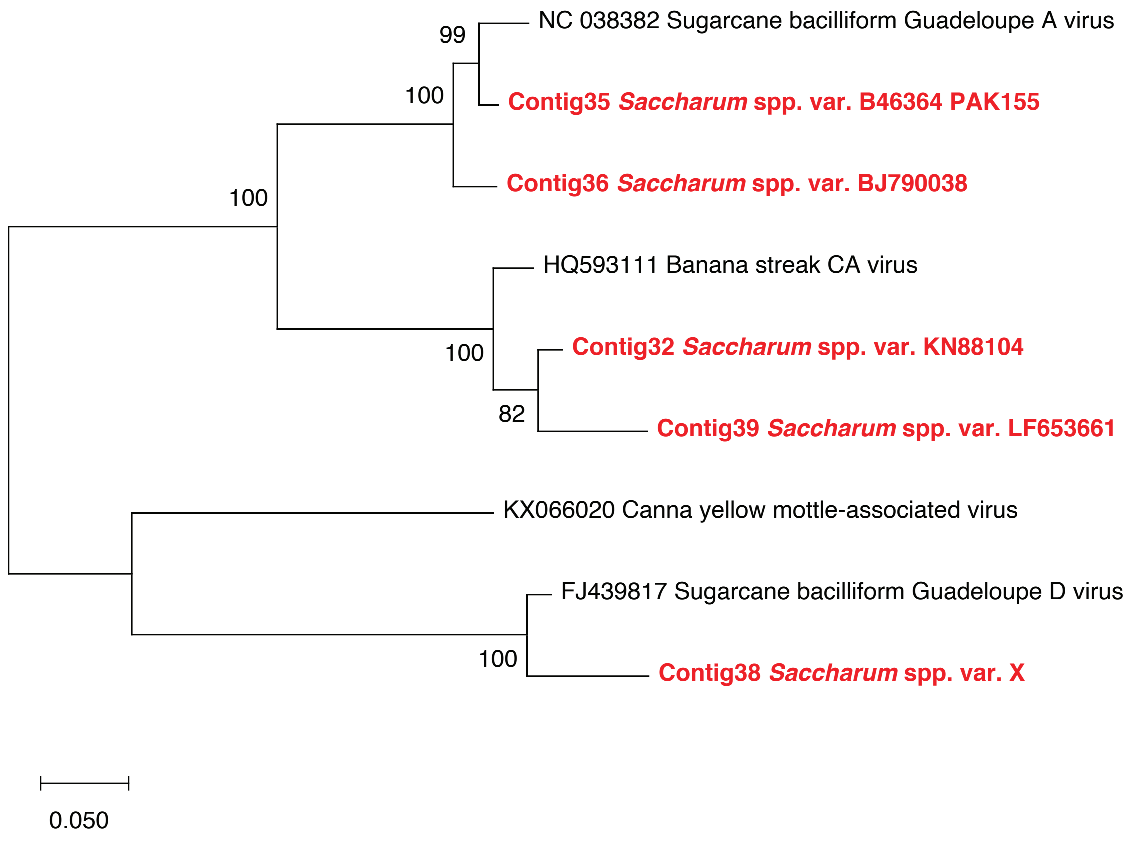

Supplement: Supplementary file 1 [file viruses-13-00922-s001.zip › Supplementary Figure S1.pdf]
