# Supplementary material for: Comparison of the Virome of Quarantined Sugarcane Varieties and the Virome of Grasses Growing near the Quarantine Station
Source: Viruses. 2021 May 16;13(5):922. doi: 10.3390/v13050922 (PMC8157134; doi:10.3390/v13050922)
Supplement: Supplementary file 1 [file viruses-13-00922-s001.zip › Supplementary Tables.pdf]

Supplementary Table S1. Primer pairs used for amplification of the partial genome sequence of the umbra-like sequence isolated from sugarcane variety BJ790038.

**Forward primer name**	**Forward primer sequence (5'-3')**	**Reverse primer name**	**Reverse primer sequence (5'-3')**	**Elongation time (EXT)**	**Amplicon size**
SCUMBRA1F	GGACGAGATCGCTGAAACTG	SCUMBRA2R	CAGGTTTTGGTCGTCGTTAG	30 sec	262 nt
SCUMBRA1F	GGACGAGATCGCTGAAACTG	SCUMBRA3R	GTTCGTATCGTGTCCGCC	30 sec	453 nt
SCUMBRA2F	CTAACGACGACCAAAACCTG	SCUMBRA3R	GTTCGTATCGTGTCCGCC	30 sec	209 nt
SCUMBRA4F	CTGTTCTGACTGCGACTGG	SCUMBRA5R	CTCGGGAGTGACTGTTTGC	30 sec	292 nt
SCUMBRA4F	CTGTTCTGACTGCGACTGG	SCUMBRA6R	CGAGAACGAACCACTCTGC	30 sec	458 nt
SCUMBRA5F	GCAAACAGTCACTCCCGAG	SCUMBRA6R	CGAGAACGAACCACTCTGC	30 sec	184 nt
SCUMBRA2F	CTAACGACGACCAAAACCTG	SCUMBRA4R	CCAGTCGCAGTCAGAACAG	60 sec	978 nt
SCUMBRA3F	GGCGGACACGATACGAAC	SCUMBRA4R	CCAGTCGCAGTCAGAACAG	60 sec	786 nt
SCUMBRA3F	GGCGGACACGATACGAAC	SCUMBRA5R	CTCGGGAGTGACTGTTTGC	60 sec	1060 nt

Supplementary Table S2. Wild *Poaceae* sample collected from grassland (GL) and sugarcane samples from the quarantine glasshouse infected by *Closteroviridae* viruses.

**Contig number_Plant species_Sample name**[a]	**Sample origin**	**Virus genus**	**Sequence length (bp)**	**Best BLASTn hit (NCBI)**	**Gene**
Contig1_*Brachypodium_phoeconides*_3-BP-4	GL, France	Closterovirus	535	YP_874184	Polyprotein ORF1A
Contig2_*Saccharum*_spp._var._Q112	Australia	Ampelovirus	1236	MN116751	polyprotein ORF1A
Contig3_*Saccharum*_spp._var._B46364-USA_51/1	Barbados	Ampelovirus	2687	MN116751	polyprotein ORF1A
Contig4_*Saccharum*_spp._var._B46364-PAK155	Barbados	Ampelovirus	833	MN116751	polyprotein ORF1A
Contig5_*Saccharum*_spp._var._BJ790038	Barbados	Ampelovirus	3309	MN116751	polyprotein ORF1A
Contig6_*Saccharum*_spp._var._X	Egypt	Ampelovirus	936	MN116751	Polyprotein ORF1A
Contig7_*Saccharum*_spp._var._LF653661	Fiji	Ampelovirus	1012	MN116751	polyprotein ORF1A
Contig8_*Saccharum*_spp._var._NA021668	Argentina	Ampelovirus	249	MN116751	P6_ORF2

[a]Only contigs from sugarcane samples that are in the same genomic region than the contig from *Brachypodium phoeconides* samples are mentioned in the table.

Supplementary Table S3. Wild *Poaceae* samples collected from the sloped land (SL) and the grassland (GL) and sugarcane samples from the quarantine glasshouse infected by *Luteoviridae* viruses.

**Contig number_Plant species_Sample name**	**Sample origin**	**Virus genus**	**Sequence length (bp)**	**Best BLASTn hit (NCBI)**	**Gene**
Contig9_*Aegilops_triuncialis*_2-AT-2	SL, France	Unclassified	723	MK440658	RdRp
Contig10_*Bothriochloa_barbinodis*_3-BB-2	GL, France	Unclassified	637	MK440658	RdRp
Contig11_*Saccharum*_spp._var._B46364-PAK155	Barbados	Polerovirus	5763	KY052166	complete genome
Contig12_*Saccharum*_spp._var._B46364-USA_51/1	Barbados	Polerovirus	5763	KY052166	complete genome

Supplementary Table S4. Wild *Poaceae* samples collected from the sloped land (SL), the grassland (GL) and sugarcane samples from the quarantine glasshouse infected by *Potyviridae* viruses.

Contig number_Plant species_Sample name	Sample origin	Virus genus	Sequence length (bp)	Best BLASTn hit (NCBI)	Gene
Contig13_*Brachypodium_phoenicoides*_2-BP-2	SL, France	Tritimovirus	9040	AIE45540	entire genome
Contig14_*Gastridium_ventricosum*_3-GV-3	GL, France	Tritimovirus	9131	NP_932608	entire genome
Contig15_*Saccharum*_spp. var._B46364-USA51/1	Barbados	Potyvirus	9646	NC_004035	entire genome
Contig16_*Saccharum* spp._var._B46364-PAK155	Barbados	Potyvirus	9325	NC_003398	entire genome
Contig17_*Saccharum*_var. _T9	China (Guanxi)	Potyvirus	9792	NC_004035	entire genome

Supplementary Table S5. Wild *Poaceae* samples collected from the flat stone land (FSL) and sugarcane samples from the quarantine glasshouse infected by mastreviruses (*Geminiviridae* family).

Contig number_Plant species_Sample name	Sample origin	Virus genus	Sequence length (bp)	Best BLASTn hit (NCBI)	Gene
Contig18_*Anisantha_madritensis*_1-BM-1	FSL, France	Mastrevirus	3554	MK546381	entire genome
Contig19_*Anisantha_madritensis*_1-BM-2	FSL, France	Mastrevirus	3683	MK546381	entire genome
Contig20_*Saccharum*_spp._var._KN88260	Sudan	Mastrevirus	2007	MK606500	*cp*, *rep*
Contig21_*Saccharum*_spp._var._R579	Réunion	Mastrevirus	3448	MK606500	entire genome
Contig22_*Saccharum*_spp._var._KN88104	Sudan	Mastrevirus	958	MK606500	*cp*
Contig23_*Saccharum*_spp._var._KN88147	Sudan	Mastrevirus	3422	MK606500	entire genome
Contig24_*Saccharum*_spp._var._KN8924	Sudan	Mastrevirus	2838	MK606500	entire genome
Contig25_*Saccharum*_spp._var._KN8843	Sudan	Mastrevirus	1277	MK606500	*cp*, *rep*
Contig26_*Saccharum*_spp._var._X	Egypt	Mastrevirus	2946	MK606500	entire genome
Contig27_*Saccharum*_spp._var._USDA	Egypt	Mastrevirus	3247	AF037752	entire genome
Contig28_*Saccharum*_spp._var._USDA	Egypt	Mastrevirus	3208	MK606500	entire genome

Supplementary Table S6. Selected contig sequences of Wild *Poaceae* samples collected from the grassland (GL) and sugarcane samples from the quarantine glasshouse potentially infected by badnaviruses (*Caulimoviridae* family).

**Contig number_Plant species_Sample name**	**Sample origin**	**Virus genus**	**Sequence length (bp)**	**Best BLASTn hit (NCBI)**	**Gene**
Contig29_*Brachypodium_phoenicoides*_3-BP-5	GL, France	Badnavirus	223	KX255726	polyprotein
Contig30_*Brachypodiu_ phoenicoides*_3-BP-5	GL, France	Badnavirus	228	KX255726	polyprotein
Contig31_*Saccharum* spp._var._R579	Réunion Island	Badnavirus	3110	NC_038382	polyprotein
Contig32_*Saccharum*_spp._var._KN88104	Sudan	Badnavirus	726	HQ593111	polyprotein
Contig33_*Saccharum*_spp._var._Q112	Australia	Badnavirus	1159	JN377534	polyprotein
Contig34_*Saccharum* spp._var._B46364-USA51/1	Barbados	Badnavirus	2935	NC_038382	polyprotein
Contig35_*Saccharum* spp._var._B46364-PAK155	Barbados	Badnavirus	6534	NC_038382	polyprotein
Contig36_*Saccharum* spp._var._BJ790038	Barbados	Badnavirus	7413	NC_038382	entire genome
Contig37_*Saccharum* spp._var._KN8843	Sudan	Badnavirus	870	HQ593111	polyprotein
Contig38_*Saccharum* spp._var._X	Egypt	Badnavirus	*7555*	FJ439817	entire genome
Contig39_*Saccharum* spp._var._LF653661	Fiji	Badnavirus	469	HQ593111	polyprotein

Supplementary Table S7. Wild *Poaceae* samples collected from the sloped land (SL), the grassland (GL) and from sugarcane samples located in the quarantine glasshouse, containing *Umbravirus* or umbra-like virus contigs. Contigs in bold were used for phylogenetic analyses.

**Contig number_Plant species_Sample name**	**Sample origin**	**Virus genus**	**Sequence length (bp)**	**Best BLASTn hit (NCBI)**	**Gene**
Contig40_*Trachynia_distachya*_2-BD-3	SL, France	*Umbravirus*	**2457**	MN868593	RdRp + partial ORF3
Contig41_*Phalaris_minor*_2-P-5	SL, France	*Umbravirus*	**414**	MK211274	RdRp
Contig42_*Phalaris_minor*_2-P-3	SL, France	*Umbravirus*	**654**	MK211274	RdRp
Contig43_*Festuca*_sp._3-F-5	GL, France	*Umbravirus*	**1658**	MK211274	RdRp
Contig44_*Festuca*_sp._3-F-6	GL, France	*Umbravirus*	**496**	MH922775	RdRp
Contig45_*Bothriochloa_barbinodis*_3-BB-1	GL, France	*Umbravirus*	347	MF415880	RdRp
Contig46_*Brachypodium_phoenicoides*_3-BP-4	GL, France	*Umbravirus*	**619**	MK211274	RdRp
Contig47_*Saccharum*_spp._var._BJ790038	Barbados	*Umbravirus*	**1931**	MF415880	RdRp
Contig48_*Saccharum*_spp._var._KN8924	Sudan	*Umbravirus*	**2041**	MF415880	RdRp
